# ‘Their opinion counts as far as you let it’- understanding queer migration in Minas Gerais, Brazil

**DOI:** 10.1080/0966369X.2023.2298790

**Published:** 2024-01-25

**Authors:** Fernanda Fortes de Lena

**Affiliations:** a Department of Demography, University of Campinas, Campinas, São Paulo, Brazil; b Centre d’Estudis Demogràfics (CED) – CERCA, Bellaterra, Barcelona, Spain

**Keywords:** Family, global south, LGB, life course, migration, sexuality

## Abstract

The literature regarding the life course and queer migration has shown that many gay men and lesbians seek large cities to live their lives away from the prying eyes of their families and build their sexual identity. In the global south, little is known about the effects that sexuality can have on the migratory trajectories of individuals. In that sense, what happens to the lives of those that have never left their hometowns and have had to find ways to experience their sexuality in these places. Therefore, the aim of this paper is to discuss queer migration, the impact on the lives of the individuals that left their hometowns, others that at one point came back and those that never left in the first place. Based on 21 life course interviews with self-identified LGB individuals in small/medium towns in Brazil, this paper shows how aspects such as closeness to family, educational trajectory, financial stability affects the migration trajectories of LGB individuals that live in small/medium cities. The results show that families are an important influence in the decision-making to migrate, to stay or to return to your hometown.

## Introduction

As migration dynamics become more complex during the twenty-first century, the mobility paradigm unfolds into a process that impacts and shapes the lives of individuals and their surroundings (Sheller and Urry 2006). In the field of geography, internal migration is responsible for the redistribution of population in a given territory. The question as to what drives internal migration has been addressed through many theories and has its roots in the nineteenth century in which labor mobility was responsible for large migration flows (Etzo 2008; Ravenstein 1980). The ‘new mobility paradigm’ according to Sheller and Urry (2006) has brought to light novel forms of analyzing internal migration in many countries including Brazil that is undergoing a restructuring of its urban landscape in the last twenty years (Brito 2015). In this context, the country has undergone changes in its migration patterns that were mainly directed to large cities and their metropolitan areas to an increase in short distance migration that are mostly interregional (Baeninger 2012; Cunha 2011; de Lima and Braga 2013). Another aspect of recent migration patterns in the country is the increase in the number of returned migrants that has a great impact in states like Minas Gerais and states in the Northeast region of the country (Baptista, Campos, and Rigotti 2012; Brito, Garcia, and Carvalho 2002). In this Brazilian context, an aspect that has few studies (Fortes de Lena 2022; França 2013) is the internal migration of sexual minorities in a country that has acquired many civil rights in the last decade such as the legalization of same-sex marriage in 2013 and the criminalization of homophobia in 2019 (Facchini and Isadora 2020). Other context in the Global South, especially in Asia have been more prolific in the studies of queer migration even though most are still about international migration (Ha 2020; Lucetta 2019; Yu 2021). In this study, I will look at studies conducted in a Latin American context that have internal migration as their focus of analysis.

Most studies that focus on migration of sexual minorities, often referred to as queer migration studies, have explored the rural-urban migration and the potential of migration for self-realization and sexual freedom (Knopp 2004; Lewis 2014; Weston 1995). Since the increase in urbanization the internal migration of sexual minorities has become more complex with some studies focused mainly on their spatial distribution in urban areas and the characterization of their surroundings (Compton and Baumle 2012; Cooke 2005; Wimark and Östh 2014). With the increase in social acceptance of homosexuality over the last two decades (Pew Research Center 2020), some scholars have turned their attention to rural areas and the lives of sexual minorities in those spaces (Gorman-Murray, Waitt, and Gibson 2008, 2012). Annes and Redlin (2012) have analyzed returned migrants that initially migrated to the city and have gone back to small rural areas later in life, showing that the multiplicity of trajectories of sexual minorities over their life course.

The aim of this article is to contribute further to the latter direction in queer migration studies by both adding a perspective from the global south and exploring how family dynamics may influence or not migration. Therefore, in this study, I focus on the life course narratives of gay men, lesbians and bisexual women in small/medium towns in the Southeast part of Brazil, in which they describe their thoughts of leaving, their reasons for staying and also the feelings of returning to their cities of birth.

### Queer migration through a life course approach

The life course approach has gained more ground in social science over the last decades for it takes into account different social, institutional and geographic contexts that may impact the lives of individuals (Elder Jr. and Giele 2009). This method not only allows for a more macro analysis of life events but also combines them with individual traits such as age, gender and ethno-racial identities making it possible to identify timing and patterns throughout the life course enabling comparisons across and within groups. The life course approach regarding gay men, lesbians and bisexuals has grown in recent years (Lewis 2014; Wimark 2016a), but there is still a lot to uncover regarding the differences and communalities of the life course of these individuals.

In the narratives of the life stories of gay men and lesbians, one life event stands out for being particular to this group: the coming out story. This experience in the life of sexual minorities has given fuel to studies that make connections between this event and others such as residential mobility, migration, educational trajectory and entering labor market (Lewis 2014; Waitt and Gorman-Murray 2011; Wimark 2016a).

In analyzing the life course of gay men, Lewis (2014) shows that migration is an important life event that not only helps to build sexual identity, but also assists young gay adults in transitions of establishing careers or becoming a part of a community. The study also identified traces of ‘coming out migration’, which Gorman-Murray (2009) had called attention to in his essay towards the search of an imagined gay community by individuals that migrate from small towns. It was also Gorman-Murray (2007) that recognizes that what is known about migration of gay men and lesbians has mostly been based on the experiences of individuals in the Global North. Adding to that, Brown et al. (2010) acknowledges the absence of analysis of sexualities in the Global South by scholars in the Global North and points to the importance of engagements that dialogue between these two spheres.

The life course approach also enables the analysis of individual agency, which forges the structure of pathways of sexual minorities. Although, limited to the opportunities that are presented according to social background and economic constraints (G. H. Elder Jr., Johnson, and Crosnoe 2003). According to Settersten (2003), age-related experiences are important in establishing the different roles expected for individuals in a specific time and place. To this end, individuals are expected to leave their parents’ home, get an education, enter the labor market, marry, form a family, and retire at different points in their lives.

In this context, Wimark’s (2016b) worked on how family ties in Turkey are important in the life course trajectories of gay men and lesbians, bringing attention to the impact of the coming out process for sexual minorities. The findings show that family can have a positive impact by being supportive in different aspects of life emotionally, financially and socially. At the same time, they can be unsupportive of the coming out process, which leads to a continued impact on the migration trajectories of these individuals.

The first studies that depicted the relationship between rural areas and sexuality originated in the field of geography, in which David Bell and Gill Valentine have been pioneers by showing the different misconceptions of ‘Queer Country’, one of them being the necessity of urban surrounding to live a ‘gay life’ (Bell 2006; Bell and Jayne 2006). Annes and Redlin (2012) explore the relationship between coming out, first same-sex experience, self-acceptance and migration from rural to urban spaces. The study focuses on four narratives of gay men that show the dichotomy of the city that allows sexual exploration, but at the same time expects a certain type of gay identity behavior. On one hand, the study finds that the city is key for identity formation and on the other the return to the country is important for a greater understanding of who they are away from the city. Thus, the life course framework enables the acknowledgment of different contexts in which sexual minorities are inserted as well as identifying the life events that are specific to them and others that they share with heterosexual individuals. This framework establishes parallels between the trajectories within the group of sexual minorities.

This study takes on this aspect of rural migration by focusing on the lives of sexual minorities that reside in small/medium towns. Therefore, I delve on the connections between family and migration using a life course perspective that sheds some light on the repercussions that this relationship can have on the decisions of gay men, lesbians and bisexuals to migrate, to return or to stay in their city of origin.

### Field site and internal migration in Minas Gerais

In this study, I focus on small/medium towns in the state of Minas Gerais located in the southeast region of Brazil. This geographical scope allowed to reflect on how places that are very religious and with limited resources regarding educational training and labor market can affect the migration trajectory of sexual minorities that reside in these towns.

The choice of conducting fieldwork in Minas Gerais is three folded. First, most studies about LGB individuals and migration in Brazil have been conducted mainly in the metropolis of São Paulo and Rio de Janeiro (Campos and Moretti-Pires 2018; França 2013; Parker 2002), and a study in another state of the same region can contribute to specific contexts as well as to establish similarities with previous studies. Secondly, the state of Minas Gerais is known for its deep-rooted Catholicism especially in small/medium towns that have historic churches as main attractions for tourism. The implications of living in an environment based on Catholicism are reflected on how people should conduct themselves in that society (Busin 2011). In this sense, religion is in the background of the decision-making of sexual minorities that reside in cities in which the population is mainly Catholic. Thirdly, Minas Gerais has an advantageous localization because of its large extension it has administrative borders with almost all other regions in the country, which are connected through roads that cross its territory making the mobility between municipalities more accessible.

**Figure 1.**
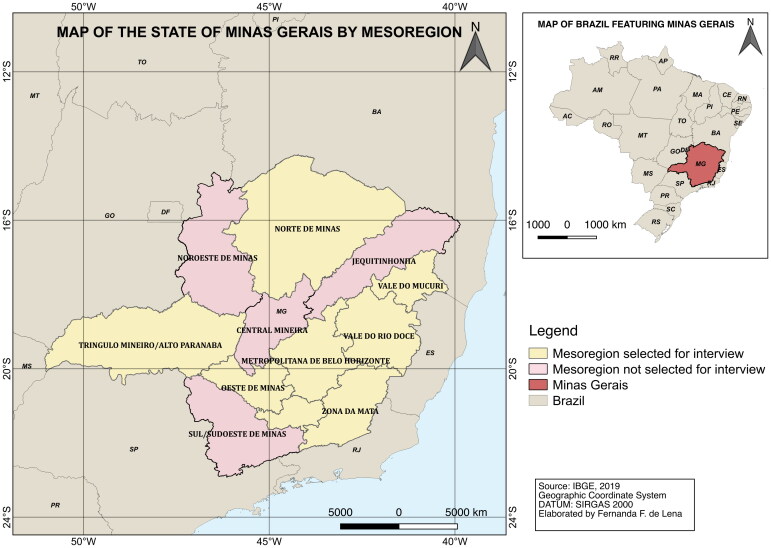
Map of the state of Minas Gerais by mesoregion, 2019. Source: Elaborated by author.

The field sites were chosen because of their geographical distribution in Minas Gerais, which is a state that has twelve meso-regions from which nine were selected, and from these nine mesoregions at least one municipality was chosen to find individuals to be interviewed (see Figure 1). The choice of the municipalities visited took a number of considerations into account. The first condition was that the municipality should be a mesoregional capital in the region. This would make it easier to satisfy the other two conditions; presence of at least one university in the municipality and population size of at least 50.000. In the end, 11 cities were visited, and 21 individuals were interviewed between July and August of 2019. The two other conditions were chosen because in recent migration literature in which there has been indications of how leaving your parents’ house to attend university in another city has become a common practice after the creation of public policies towards the expansion of higher education in the country (Lucchesi and Saad 2007). Therefore, having a university in the city could open the possibility of staying in the city to study or leaving to attend another university. In this context, this situation enables the discussion related to the individual agency of leaving or staying in the city of origin. Adding to that, cities with a minimum population size of 50.000 opened the possibility of finding individuals willing to be interviewed and making sure that their identity remained anonymous and protected during the interview.

In this section, I also give an overview of the internal migration in Minas Gerais to situate the migratory trajectories of the participants in a more general context as part of a larger migration process that has been occurring in the last few decades. According to Filho and Bueno (1976), there has been a historic migration to the medium cities in the state of Minas Gerais that has a labor market that can absorb individuals from small towns and rural areas, which eventually interrupts the migration trajectory of these migrants that were initially directed to more populated cities. This flow to medium cities is still present in the migration of some participants, but instead of migrating to look for jobs the main reason in the case of most participants is the search for higher education. This is due mostly to the expansion of federal universities in medium cities in Minas Gerais (Martins et al. 2022).

With the changes in migration patterns in the country, return migration became more common and some scholars were interested in measuring the impact of this type of migration in the population of Minas Gerais (Garcia and de Miranda-Ribeiro 2005; Lobo and Matos 2017). Recently, Carvalho and Rigotti (2015) studied the impact of migration on the population growth of medium cities in Minas Gerais. The findings show that most medium cities benefited from in-migration, which was responsible for a large part of the population growth in medium cities in the last decade. The conclusions point to the importance of young in-migrants in slowing down the aging of the city’s recipients of those migrants and that the medium cities that had negative net migration were aging faster.

Therefore, in a general context Minas Gerais is presented as a state that was historically losing population *via* out-migration to other states (São Paulo, Rio de Janeiro and Brasília) and became a state that is retaining population and receiving returned migrants in the last couple of decades (Garcia and de Miranda-Ribeiro 2005; Lobo and Matos 2017).

### Study design and method

In this study, a life course perspective is used where events in the individuals’ life are highlighted through life course narratives. The life course interviews were conducted tracing parallels to the participant’s life events focusing on understanding their sexuality, coming out, school life, migration, relationships, return to city (when it occurred), financial independence and networks. All these events have been shown to influence the trajectories of these individuals that can differ from the life course of non-LGB individuals (Hammack and Cohler 2009). The life course narratives were collected through a method of life story narratives that highlighted the relationships to family, friends, and the city (Atkinson 1998).

The selection of participants was based on the snowball method with an initial contact through a wider social call in my personal LGBT circles in the cities that filled the requirements to be chosen (Figure 1). This approach to finding people to interview was reflected in the age range (21 to 35) I was able to engage with in the municipalities I had chosen. The personal network I reached out to find the participants in different municipalities was also reflected in finding mostly participants with a university diploma or undergrad students given my own higher education background. This was a limitation since there were not participants with very different educational levels. Although, this homogeneity educationally was interesting to analyze showing the different paths that were taken in pursue of higher education.

The research project underwent an ethical review by the Ethics Committee through the University of Campinas (no. 18674519.7.0000.8142). The interviews lasted between 50 min and 2 h and 45 min and took place in locations of choice of the participants and convenience of the interviewer. The interview was composed of an initial questionnaire of sociodemographic characteristics of the participants and a semi-structured script was used to ensure that most topics were covered by the interviewer and the participant (Appendix I). The information on that questionnaire was later aggregated to Table 1. Since the participant’s self-identified as lesbian, gay or bisexual during the interviews I maintained this classification that was spontaneously given during the narratives (see Table 1).

**Table 1.** Sociodemographic characteristics of the participants.

Age	Gender	Sexual Identity	Educational level	Race/ Ethnicity	Civil status	Migratory status
23	Female	Bisexual	Undergrad	White	Single	Migrant
25	Female	Bisexual	Undergrad	White	Married	Migrant
21	Female	Bisexual	Undergrad	Black	Single	Migrant
34	Male	Gay	M.A	White	Single	Migrant
25	Male	Gay	Bachelor	White	Single	Migrant
27	Male	Gay	Undergrad	White	Single	Migrant
27	Female	Lesbian	Undergrad	White	Single	Migrant
25	Male	Gay	Undergrad	White	Single	Non-migrant
29	Male	Gay	Bachelor	Pardo	Single	Non-migrant
25	Male	Gay	Undergrad	Pardo	Single	Non-migrant
22	Male	Gay	Undergrad	Black	Single	Non-migrant
34	Male	Gay	Bachelor	White	Single	Non-migrant
35	Male	Gay	Bachelor	White	Married	Non-migrant
31	Female	Lesbian	Bachelor	White	Single	Non-migrant
25	Male	Gay	Undergrad	White	Single	Returned
35	Female	Lesbian	Bachelor	Pardo	Single	Returned
28	Female	Lesbian	Bachelor	White	Single	Returned
28	Female	Lesbian	Undergrad	Black	Single	Returned
28	Female	Lesbian	Bachelor	Black	Single	Returned
30	Male	Gay	Bachelor	White	Single	Returned
31	Female	Lesbian	M.A	Pardo	Single	Returned

Source: Elaborated by the author.

The interviews were transcribed and coded with the migratory trajectory, as the main category of analysis (Creswell 2014). If the participant was a non-migrant then the focus was on thoughts of leaving and the reasons related to staying in their hometowns. Afterwards, sub-codes were created according to the themes that were interesting and related to migrations like coming out, moving to study, coming back, thoughts of leaving, etc. There was a multiplicity of narratives that were quite diverse which are not all described in this study for it would defy its purpose. Nevertheless, three main themes were identified: coming out, educational trajectory and financial dependence. I analyze these themes separated mainly by migratory status: migrants, returned and non-migrants. Inside each group I consider the compositions by age, gender and race/ethnicity.

## Migrants, stayers and returned

In migration studies, the focus on the trajectories of the migrants can help explain mobility across borders by identifying the mechanisms that can influence the migratory decision-making. On one hand, the non-migrants (stayers) are mainly neglected in migration studies, but can also inform the migratory phenomenon since they are what can be called the control group. On the other hand, there are the returned migrants, which create a reverse migratory flow back to the city of origin that is also an important point to understand mobility and the demographic impact of that mobility on the receiving population. Therefore, the participants in this study were divided into three groups of migratory status: migrants, non-migrants (stayers) and returned migrants. Of all the participants, only four were born in a municipality, but moved with their parents to another municipality at a very young age. This mobility was not considered in the final categorization in Table 1, since all these four participants lived their childhood and adolescence in this second municipality with their parents. The non-migrants were those individuals that had never moved to another city for longer than six months. Lastly, the migrants were those who had left their town of birth having at least 17 years of age and were now living in another municipality. Therefore, unintentionally I interviewed 7 migrants, 7 non-migrants and 7 returned migrants.

## Results

After a careful analysis, I was able to identify three main themes related to the relationship to family and migration that unfold in the following sections: disclosure of sexuality, educational trajectory and financial dependency of the participants. The first theme, disclosure of sexuality, is a common thread among the narratives of sexual minorities (Lewis 2014; Waitt and Gorman-Murray 2011; Wimark 2016b) and can be classified as a life event that leads to a turning point in the life of an individual, affecting the other themes. The second theme, educational trajectory, is important in the life course of most individuals and understanding the negotiations that happen between the participants and family members is essential to the development of these trajectories. The third theme, financial dependence, is the force that holds some participants captive to their parents’ expectations. At the same time, financial independence can release the tensions between the disclosure of sexuality and family ties. In these sections I analyze how those experiences can be different for the three groups: migrants, non-migrants (stayers) and returned migrants. Another characteristic that intersects this analysis is the gender of the interviewed in which most of the non-migrants were gay men (6). This was something that came up during one of the interviews where I asked Ivo, a returned migrant, gay, 25 years of age, living with his mother, that said that most of his lesbians’ friends had left the town, but his gay friends remained there. I asked why he thought that, and his response was: ‘Courage! They are braver than us! The gay man to step out from underneath his mother’s wing (…) I think it’s a question of bravery! We (gay men) wait for the need to leave our parents’ house, women have the desire to leave.’

In this sense, while most ‘stayers’ were gay men, the returned migrants were mostly women (5). The returned gay men also come back because of economic reasons, but have inserted themselves in the labor market of their hometowns with plans to leave to go to another city. This brings up questions of gender labor force inequalities that are not the scope of this research, but undeniably would be interesting to pursue in future research.

### (Non-)disclosing sexuality in small/medium towns

In the life course of sexual minorities it has been identified that the disclosure of their sexuality to family, or commonly known as ‘coming out story’, is considered to be not only a life event, but also a turning point for some individuals (Hammack and Cohler 2009). However, not all individuals disclose their identity to their family and there are different strategies and outcomes to this life event (Lewis 2014; Wimark 2016b). In this section, the focus is given to unfolding of the disclosure or the non-disclosure of the participants sexuality and the family’s reaction. The aim with this is to establish if family support has some influence in the decision-making of the participants that decide to leave or stay in their hometowns. In analyzing the narratives of the coming out process of the participants and the reaction of the families a few points need to be taken into consideration. The first is how the disclosure of the sexual orientation is communicated to family members. The second is the different kinds of support that families offer to the participants. Finally, how does disclosure or non-disclosure come together and influence the migration trajectories of the participants.

The ‘coming out’ story in queer migration studies in the global north, in which migration and disclosure of sexuality have been shown to be intimately related are an important starting point in the migratory trajectories (Bell and Valentine 1995; M. P. Brown 2000; Knopp 2004). Also important in queer migration is the non-disclosure of sexuality to family, in which migration is used as a buffer between them and their family (Wimark 2016b). Boussalem (2021), shows a situation in between where a ‘tacit knowledge’, a non-verbal understanding of the sexuality of the participants that was used to convey an acceptance without having to make it explicit. All these scenarios come to show that there are different strategies in how to communicate sexuality outside a binary in/out the ‘closet’ as depicted in western societies.

In this study, it becomes clear that in a Global South context there is also not a fine line between the act of disclosing/non-disclosing and how people navigate them towards family members. Even though, most of the participants have disclosed their sexuality to their parents at some point, the family reaction varies across the participants. The most common response is the silence surrounding the relationship between family and participants concerting their sexual orientation, which is a mechanism used by the parents of not talking about their sons/daughters sexuality after their disclosure as a form of denial and/or non-validation of that part of their lives (Nascimento and Scorsolini-Comin 2018; Oliveira and Barreto 2019). As Leandro, 22, non-migrant, talks about his relationship with his father after he disclosed his sexual orientation to him ‘Although I think he doesn’t like it, he never gave me problems, you know? And on the same day we continued our lives as if nothing had ever happened. I mean, we never talk about it.’

In some cases, the non-disclosure of sexual orientation by a minority of the participants was met with fear of confrontation and was justified mainly by the lack of need to disclose this information to loved ones since they thought in some way it was implicit in their life choices. The narrative by Túlio, 34, non-migrant, shows parallels to the ‘tacit knowledge’ identified by Boussalem:

My father doesn’t know about my sexual orientation, but like… he doesn’t know… like from my mouth, but I’m sure that other people have said something to him in that sense, but until this day he never had the curiosity of asking me. (Túlio, 34, non-migrant)

As noted in other studies family support is essential to the participants in their decision-making to leave or stay in their hometowns (Lewis 2014; Wimark 2016b). On one hand, the participants that have more accepting parents tend to stay and course university while living with their parents. On the other hand, the non-accepting parents are also non-supportive. Unfortunately, having accepting parents is the least common scenario among the participants, like in the case of Leonardo, 25, non-migrant, recalling what his mother said after he told her he was gay *Are you sure about what you told me? I said yes and she responded: Well, in that case it is going to be hard for us to live together then. I said ok and packed my things and asked my uncle if I could stay with him for a while. He didn’t ask me anything, but he knew what was going on*. In this case, Leonardo didn’t leave his hometown because he was already studying at the university and had support from other kin, which helped him stay.

The migrants that didn’t disclose their sexuality to their parents also saw this as an unnecessary event that would not change their situation like when Emerson, 34, migrant, talks about why he didn’t tell his family:

I have always been able to live my sexuality this way, you know? Even after I moved here and I am very free in that sense. I don’t need to talk to my mother always, you know? There is no need for me to tell them for me to be free in this city.

The narratives of partial disclosure or the ‘tacit knowledge’ of the sexuality of the participants influence the way in which the family supports the decision to migrate of these individuals. For some women that had disclosed their sexuality, the disbelief from the parents that they would not leave shows a gendered perspective into the support families give to lesbian and bisexual women that want to migrate:

I was studying to get into college, but they (parents) didn’t have faith that it would happen. They thought I wouldn’t have the courage to leave and live by myself.’ (Roberta, 25, migrant)

The dynamics between the participants and their families is guided not only by the disclosure of their sexual orientation, but by the negotiations that occur after the disclosure (Lewis 2014). At the same time, the limits set by how much support the family is willing to give that person informs their decision to leave the city they grew up in or continue living there.

Therefore, I show that in the Brazilian society in which family is at the core of social structures, the negotiations and strategies of living with family are diverse as represented Joaquim’s mother that will allow him to date men as long as he has a girlfriend to maintain appearances. This type of negotiation is unthinkable in other conservative societies as shown by Wimark (2021).

In short, the relationship between the family support and the sexual identity of the participants has a big effect on the participants decision to leave because most want to stay away from their non-accepting families. This is the case in other studies in the Global North (Boyle, Halfacree, and Robinson 1998; Wimark 2016b) and continues to be a driver for queer migration in the Global South. Another finding is that most participants that had accepting families decided to stay in their hometowns and those that experience the ‘tacit knowledge’ are left with the decision to stay and continue with this relationship living in the same town. For those participants, the influence of the family in the migration is intertwined with other aspects of the migration process that I touch with more detail in the next sections.

#### Education as a gateway for independence and identity building

Educational mobility in Brazil has become more common after the implementation of public policies that were put in place to support students that come from a socially disadvantaged background (Souza and Almeida 2019). The group of participants that have left their hometowns in pursue of higher education can be characterized as mostly bisexual women and gay men that migrated at a young age between 17–20 years. Only two of them came from cities that had universities, the other five came from very small towns with no possibilities of continuing their studies.

In their narratives, wanting to leave to advance in their studies was seen as a common path as Karina, 23, white, stated ‘*I said I wanted to leave…There were not many opportunities there. (…) So I had to leave if I wanted to study*.’ Other participants saw the opportunity to leave to study as a means to an end, which was always to leave their hometown:

It was through a course that I managed to find space to say: I’m going to move out! I think that now I have a reason to go, right? I seized the opportunity and came. (…) I was accepted into the university with a scholarship and said: this is where I’m staying until I get my college degree and all. (Alexandre, 25, migrant)

As Alexandre stated later that getting the scholarship at the university was determinant for his stay in the city to continue his studies or else, he would not have been able to afford it. Although, the objective of the participants was to leave to study the reasons for the decisions of each participant varied quite a lot. In some cases, like Joana, 21, black, having family members in the city of destination made her parents push her to decide to go to university there, even having been accepted in another university in the capital. ‘*My parents said it was too big (the capital), but I also liked coming here*.’. Another participant, Luciana, 27, migrant, says that the choice of leaving had mostly to do with where she could find a city that had a labor market she could be inserted and manage to support herself while studying:

I was accepted into the university here and [alternative city of destination]. But because I needed a job, I thought [city of destination] looked like a city with more commercial activity, of services, that didn’t just revolve around the university.(…)So I made a choice and chose [city of destination] at the time. (Luciana, 27, migrant)

These findings show that the places these individuals are found after migrating are regarded as the best option in the transition of finishing high school and entering university. The family in the role of an economic support was certainly not the case for all participants and this clearly affected the choices made by participants that had to think of economic strategies that enabled them to stay in the city to study. A few of the participants were clear about their alter motivations of leaving their hometowns and both participants that related the move to their sexual identity were gay men. The bisexual women only started self-identifying as bisexual once they were in the university environment and started dating women. Therefore, for these participants the migration is part of the identity building of these women since before migrating this identity was not as clear to them as it became afterwards, different from the gay men that recollected knowing they were gay from a very young age.

Migration can have an initial motivator such as the search for education, but can have different effects in the life course of individuals related to their sexual identity building. This embodied sexuality mobility is referenced in Knopp (2004) as a ‘quest for an identity’ and in this case the migration was motivated by educational purposes and the environment help Roberta realize her bisexual identity, which wasn’t clear to her even having had homosexual experiences before the migration:

It was in (Name of the city) that the key turned because I was living in a more alternative space, very cool. And I found out that I didn’t like just masculine girls. There I liked **girls** (…) It was a discovery for me that I liked women and men. (Roberta, 25, migrant)

In this section the role of family in the decision-making regarding migration to obtain higher education is shown to be related to the economic support that families can give as well as the lack of economic support of families. In the latter case, public universities play an important role of providing scholarships and financial aid for students with low-income families. The participants that are unable to access financial aid in public universities are drawn to the labor market of the city of destination.

The decision to migrate in search of education is not restricted to LGB individuals in Minas Gerais as Lopes (2008) has shown in her study with individuals that migrate to the capital (Belo Horizonte) to obtain education at a higher level. The difference in the decision to migrate in this study to the migration studied by Lopes (2008) is that most gay men in this study have a second motivation for migrating that relates to their sexuality and the need to leave the city and their parents’ home to live more freely. This result is in tune with the findings of other studies in the Global North (Annes and Redlin 2012; Lewis 2014; Wimark 2016b). It also shows that economic support from family is an important factor in the decision-making of where to migrate for these participants. Therefore, the educational trajectory of these individuals isn’t one-sided, but rather relational, in which they consider the relationships to family, financial resources and the availability of educational resources in the city of origin in their decisions.

#### The curse and relief of financial dependency

An important theme that came up during the interviews was how financial independence was a goal for most of the participants and how the relationship to their parents was changed by the transition from a dependent to an independent son or daughter financially. The monetary hold that parents have on their sons/daughters can influence the disclosure of their sexual identity because of the limitations that they can impose to the accomplishment of this financial independence by not investing in their children’s education. Another way to perceive it is through financial independence comes social acceptance by the parents:

Because an important thing also was not needing my mother’s money. Our relationship changed from water to wine. Actually, today it’s she who owes me money. (J, 27, migrant)

I think that the main thing for me to come out to my family is me having my own money to support myself. Because what holds me down now is that I depend on my father. (Joana, 21, migrant)

In many ways the financial dependency can be a trigger that sets in motion a series of events that make gay men and lesbians who feel unwelcomed in their parents’ home come up with strategies to leave. In some cases, they are asked to leave after disclosing their sexuality and are forced to become financially independent at an early age. After his main financial provider, his mother, had asked him to leave, Leonardo turned to his uncle, a possibility that for Leonardo was determinant for him to stay in his hometown and continue attending college and remain close to his sisters, which he would visit every two weeks on the weekends.

The return migration of rural gay men after living in the city weren’t economically related as most return migration argue are the main drives of this type of migration. Annes and Redlin (2012) show that the rejection of city environment regarding the gay scene found by the participants were not what they wanted for them.

The returned migrants are older than the groups of migrants since they are in a different phase of their life course. The migrants are mostly college students and the returned migrants have obtained their diploma recently in another city, but were not able to transition into the labor market and therefore, had to resort to moving back in with their parents. Another situation found are those participants that because of financial difficulties had to leave college and return to their hometown.

The complaints of the returned migrants regarding moving back to their parents’ house had mostly to do with freedom and space they had gotten used to once they left. Although, there were participants that pointed out the problems of living in the same town as their parents by being LGB and how that affected their own behavior.

If you are living here in (name of the city), you will be close to your parents. In a way you will not do things because of your parents, you know? Someone knows your father… has a family history. Because when you go to a different city you are Bianca and that’s it. Here in (Name of the city) I am Bianca da Silva Pereira, daughter of Maria and João, you know? For them it’s like a clan. It is a clan, because you need to be known by someone to probably get a job. (Bianca,28, returned migrant)

Another situation was Laura, 35, white, that had left her hometown to escape her family, but after a while her parents asked her to move back and she agreed as long as they paid for her studies:

I came back to finish my studies. The moment I finished college I was going to leave because this city is a city where people still have a narrow way of thinking. (…) But with time I adapted, I positioned myself and understood what was important, in truth; it’s not about being homosexual. It’s about being a person with opinions, that I have the right to speak (…), being a lesbian started to be a mere detail and not the main thing. (Laura, 35, returned migrant).

The use of migration as a tool to negotiate access to education can be beneficial to making financial independence a goal that can enable the participants to leave their parents’ house. Although, as Laura stated this coming back had its price of enduring living with parents that didn’t accept her sexual orientation.

So, when I bought my house, bought my things, put everything inside it, got dogs and took them to the house, I started living my life (…) And they started to understand that: ‘Well, there’s no other way, we are going to have to start understanding and respecting her because if not…’ They would lose their daughter! (Laura, 35, returned migrant)

Different from Sayad (2000), most of the participants that returned don’t see the return as a dream, but as a temporary situation that will end once they find financial stability to move out. Some of them are content in living in the same city as their parents, but those are the participants that have families that were more accepting of their sexuality when they disclosed it to their parents:

I would stay because it’s convenient, you know? I don’t have to pay rent, my family is pleasant, but I like living by myself. But if I found a job here I would stay, but I wouldn’t live with my parents. I miss the freedom of before when I lived by myself. (Bianca, 28, returned migrant)

In the study conducted by Wimark (2016a) in Malmö, the author finds that education is used as a strategy to insert themselves in the labor market, but for younger cohorts it is used as a delay for entering adulthood. In the case of the returned migrants the difficulty in entering the labor market after getting their diploma has made them resort to returning to their parent’s home. This can be considered almost as a forced delay of transition to adulthood and the participants reaffirm the temporary position of their living arrangements with their parents.

Therefore, this section shows more explicitly the links between the family financial dependency and the decision-making of participants to migrate. This was also explored in the other two sections about disclosure of sexuality and educational trajectories. However, here the financial independent participants show that having economic resources to support themselves can help the negotiations regarding the acceptance of their sexuality by family members, which makes it understandable how most of them seek this strategy from a young age.

## Discussion

Studies from the Global North have shown that gay men and lesbians migrate leaving their parents’ home in search of other cities to build their lives (Annes and Redlin 2012; Lewis 2014; Wimark 2021). This study has shown likewise that family is at the core of the decision making of most participants independently of their migration status. Like most Catholic countries, Brazil also has family as a central institutional pilar that must be protected at all costs and the same can be said about the state of Minas Gerais, one of the most Catholic states in the country. Therefore, this study can be used as a benchmark for queer migration within Brazil and future studies in Latin America that are most needed in this field of studies.

The main interest was to identify in the life course of sexual minorities in a Global South context how sexuality operates to influence individuals’ immobility or mobility. The literature has shown the importance of migration to the lives of sexual minorities that live in very small towns and feel the need to leave those places to free themselves of the oppression of family and the conservative moral expectations of the population resident (Gorman-Murray, Waitt, and Gibson 2008; Lewis 2014; Waitt and Gorman-Murray 2011; Wimark 2016b).

The relationship of the participants with their family is, to some extent, intertwined with the life events (educational attainment, entering labor market and disclosing sexual orientation) analyzed in the trajectories of these individuals. How the family reacted to the sexuality of the participants being disclosed and, the dynamics of that relationship with their next of kin. These actions and reactions influence the mobility and immobility of the participants. Not only, because some are put in movement by the family’s non acceptance, but also it sets the scene for what kind of relationship the participants will build with their families over the next years in the aftermath of this major life event.

Among the returned migrants, the main driver for their return was financial dependency. Another common narrative was the participants that wanted to pursue a different professional trajectory. Therefore, family support is important in understanding the decision-making process of returning to their hometowns.

The structure of the educational system in Brazil is reflected on the educational trajectories of sexual minorities that differ from those in the Global North. It shows that public policies regarding access to higher education in the country have allowed many of the participants to migrate and remain in their city of destination until they obtain their degree with the scholarships and/or financial aid within the university that in many cases awards low-income students with housing and free meals at the university restaurants. Another point is the instability of the labor market that made most participants vulnerable to returning to their parents’ home or in fewer cases unable to continue their studies with those participants attending private and not public universities.

In terms, the sexual minorities that remain in these small/medium towns are faced with the choice to live and express their affection for someone of the same-sex only in private spaces. They are also faced with the choice of maintaining a relationship with their parents by allowing them to have a ‘tacit knowledge’ of their sexuality and their romantic relationships.

Lastly, the findings in this study show that migration of sexual minorities have parallels with other studies in the Global North, in which the distance from family, the educational migration and the financial independence are important to identity building and well-being of the participants (Wimark 2016b, 2016a). The differences found in this study when compared to the studies in the Global North are mostly related to economic context and educational policies that play a role in the lives of the participants with low-income families. This result shows that there a specific structural context that need to be taken into account in the Global South in queer migration. At the same time, there are commonalities in the life course of sexual minorities and their relationship to family that go beyond geographical location and cultural background.

## Appendix I. Semi-structured interview script

### Family

**Table:**

Tell me how it was growing up in [name of the town]. Tell me about you and your family. Do your parents know you are [sexual orientation]? Did you tell them? How did that go? How old were you at that time? Who else in your life know your [sexual orientation]? *Why haven’t you told your parents?*

### Education/work

**Table:**

Tell me a little about your school life. Did you suffer bullying? Do you recall other kids suffering bullying? Did you tell your parents? Did they know about the bullying? Were you openly [sexual orientation] in college? Were there other LGBT people in your class? *If went to college in another town ask: Did going to college in another town have to do with your sexual orientation? In your work environment are you open about your sexual orientation? Have you suffered any discrimination at work for being [sexual orientation]? Have you had trouble finding a job for being [sexual orientation]?

### Migration

**Table:**

How did you come to life in [name of the town]? Have you lived or visited other cities? Where were the spaces of socialization in your town? Were these places exclusively LGBT? Do you still go to these spaces? How would you characterize these spaces? Tell me how you see your town related to the LGBT community? DO you think your city is a good place to live being LGBT?

### Historic context

**Table:**

Was there any historic event or celebrities that were LGBT that were important to you growing up? Did you have any LGBT person that was a reference to you in your childhood/ adolescence? Do you think there has been change regarding discrimination against GBT in your town??

### Sexuality

**Table:**

Do you recall when you recognized yourself as [sexual orientation]? Do you remember who was the first person you talked about being [sexual orientation]? Have you had romantic relationships? Tell me about the most important ones to you. Do you feel a part of the LGBT community? What do you think you have learned from the LGBT community? What is the best thing about being LGBT? Do you go to therapy? Would you like to go to therapy?

### Networks

**Table:**

Tell me about your group of friends. Who was the most important to you? Were your friends in your adolescence also LGBT? Did you tell your friends about your sexual orientation and what was their reaction? Currently are most of your friends LGBT too? Would you say your closest friendships were made before or after you recognized yourself as [sexual orientation]? From your friends that are LGBT, are most of them still living in [name of the town] or have they moved?
